# An attenuated Lassa vaccine in SIV-infected rhesus macaques does not persist or cause arenavirus disease but does elicit Lassa virus-specific immunity

**DOI:** 10.1186/1743-422X-10-52

**Published:** 2013-02-12

**Authors:** Juan C Zapata, Bhawna Poonia, Joseph Bryant, Harry Davis, Eugene Ateh, Lanea George, Oswald Crasta, Yan Zhang, Tom Slezak, Crystal Jaing, C David Pauza, Marco Goicochea, Dmitry Moshkoff, Igor S Lukashevich, Maria S Salvato

**Affiliations:** 1 Institute of Human Virology, University of Maryland School of Medicine, 725 West Lombard Street, Baltimore, MD 21201, USA; 2 Virginia Bioinformatics Institute at Virginia Tech, Blacksburg, VA 24061, USA; 3 Global Security Directorate, Lawrence Livermore National Laboratory, 7000 East Ave, Livermore, CA 94550, USA; 4 New address for ISL: NIH National Regional Biocontainment Laboratory, 950 N Hurstbourne Pkwy, Louisville, KY 40222, USA

**Keywords:** Lassa fever virus, Vaccine, Macaque, SIV-infected, Genomic profiling, Disease markers

## Abstract

**Background:**

Lassa hemorrhagic fever (LHF) is a rodent-borne viral disease that can be fatal for human beings. In this study, an attenuated Lassa vaccine candidate, ML29, was tested in SIV-infected rhesus macaques for its ability to elicit immune responses without instigating signs pathognomonic for arenavirus disease. ML29 is a reassortant between Lassa and Mopeia viruses that causes a transient infection in non-human primates and confers sterilizing protection from lethal Lassa viral challenge. However, since the LHF endemic area of West Africa also has high HIV seroprevalence, it is important to determine whether vaccination could be safe in the context of HIV infection.

**Results:**

SIV-infected and uninfected rhesus macaques were vaccinated with the ML29 virus and monitored for specific humoral and cellular immune responses, as well as for classical and non-classical signs of arenavirus disease. Classical disease signs included viremia, rash, respiratory distress, malaise, high liver enzyme levels, and virus invasion of the central nervous system. Non-classical signs, derived from profiling the blood transcriptome of virulent and non-virulent arenavirus infections, included increased expression of interferon-stimulated genes (ISG) and decreased expression of COX2, IL-1β, coagulation intermediates and nuclear receptors needed for stress signaling. All vaccinated monkeys showed ML29-specific antibody responses and ML29-specific cell-mediated immunity.

**Conclusion:**

SIV-infected and uninfected rhesus macaques responded similarly to ML29 vaccination, and none developed chronic arenavirus infection. Importantly, none of the macaques developed signs, classical or non-classical, of arenavirus disease.

## Background

Lassa hemorrhagic fever (LHF) is a rodent-transmitted disease that causes more than 300,000 human cases per year in the West African endemic area. Mortality rates are between 15-20% in hospitalized individuals, and reach 50% in epidemic episodes [1,2]. Those who develop effective antiviral immunity survive, while those with uncontrolled high viral loads succumb [3-5]. Lassa virus (LASV) replicates in almost all human tissues with the highest titers being found in liver and secondary lymphoid organs. However, the extent of tissue damage is not enough to implicate the failure of any one organ as the cause of death [1,6]. Besides viremia, other signs of LHF include myocarditis, pulmonary edema, and in advanced cases, hemorrhage and hypovolemic shock resulting from vascular leakage [6]. Due to the undifferentiated symptoms at the onset of LHF, the opportunity to administer ribavirin treatment is often missed; so the best public health option in endemic areas would be vaccination.

Many LHF vaccines have shown promise in small animal models: viral sub-particles, peptides, DNA vaccines and viral vectors [7]. The most effective candidates have been attenuated vectors that protected against LASV challenge in primate models: vaccinia virus expressing LASV glycoprotein (GP) and nucleocapsid protein (NP) [8], vesicular stomatitis virus expressing LASV GP [9], and the ML29 reassortant vaccine expressing the GP and NP of LASV and the Z and L proteins of Mopeia virus (MOPV) [10-13]. A yellow fever vaccine, YF17D, expressing LASV GP (YF-LASV-GP) was protective in guinea pigs [14] but not in primates (ISL unpublished). ML29 is the only Lassa vaccine candidate that elicited strong cell-mediated immunity before challenge and that provided sterilizing immunity in several animal models [7,10-13]. For all the live attenuated vectors, safety concerns remain to be addressed before launching clinical trials.

One safety concern is that the Lassa endemic region of West Africa has high seroprevalence for infection with Human Immunodeficiency Virus-1 (HIV): 5-12% of young adults are HIV positive [15] with most of these individuals being unaware that they are HIV-infected. Several vaccine studies have attempted to model vaccination of HIV-infected individuals by delivering attenuated vaccines to immune-deficient animals: a Rift Valley Fever vaccine was given to immune-deficient mice [16], an Ebola vaccine (VSV-EBOGP) was given to SHIV-infected monkeys [17], and a smallpox vaccine was given to SIV-infected monkeys [18]. Two of these studies selected viremic monkeys with low CD4 counts. Monkeys for our study had been infected with a pathogenic stock of SIVmac251, and vaccinated with VSVgag that doubles the ordinarily one-year lifespans of SIV-infected macaques [19], thereby prolonging the time available for the study.

The Mopeia/Lassa (ML29) reassortant virus used in this study can be an effective, broadly cross-reactive Lassa vaccine [7,11,20,21]. The large (L) genomic segment of ML29 is derived from the mild MOPV [22-25]. The S segment of ML29 encodes the NP and GP gene products derived from LASV [10-13,26,27]. ML29 appears to be even more attenuated than its parental MOPV both *in vitro* and *in vivo*[10,11].

In this study, we employed SIV-infected macaques as a model for persons living with HIV. Our goal was to determine whether ML29 is safe and immunogenic in macaques during advanced stages of SIV-infection.

## Results

### Rhesus macaque survival and viral loads

**Figure 1:**
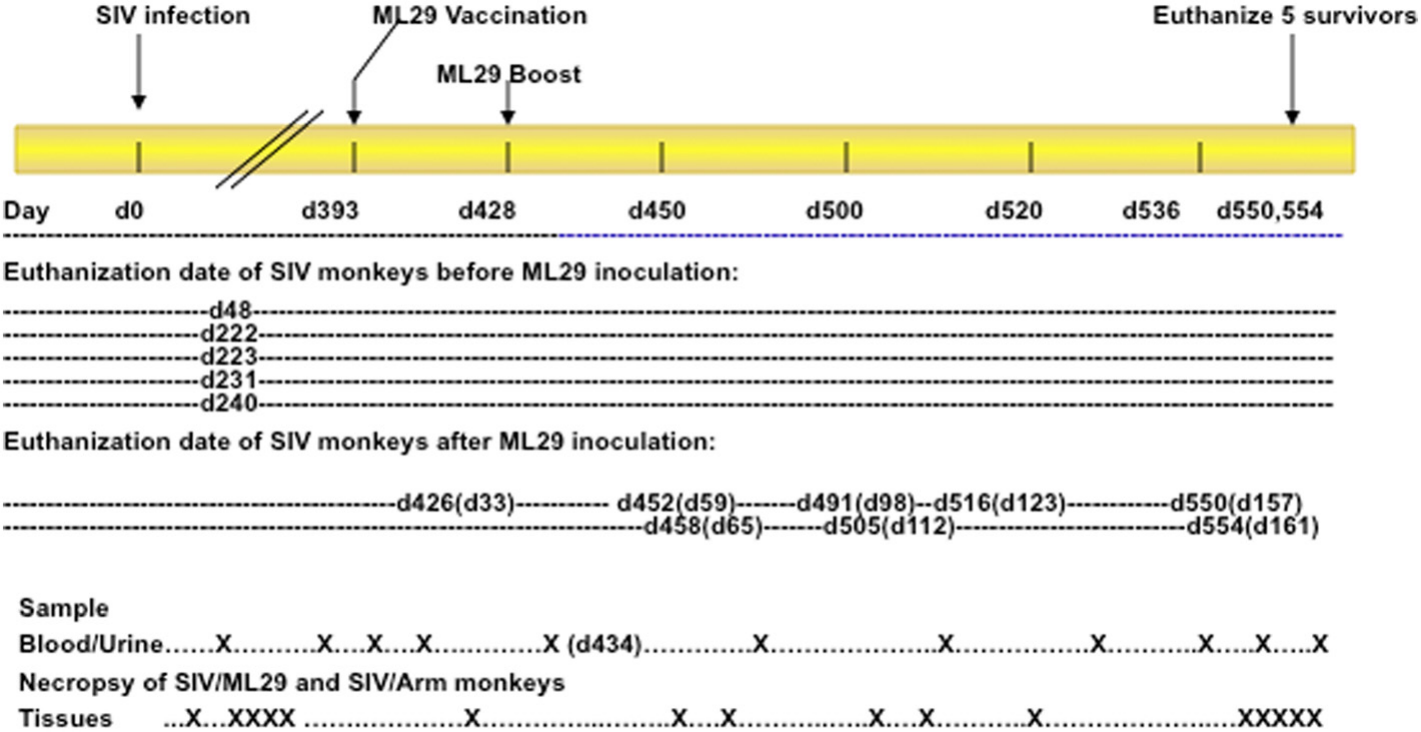
**Experimental design.** Sixteen rhesus macaques were inoculated with SIVmac251 and monitored for viral loads and SIV-specific immunity. Five succumbed within a year after SIV inoculation (48, 222, 223, 231, and 240 days after SIV infection) and the remaining 11 monkeys were vaccinated with ML29 or LCMV-Armstrong on day 393 after SIV-infection. A month after the vaccination, five monkeys (ML-1, SIV/ML-1, SIV/ML-2, SIV/ML-6, SIV/ML-7) were boosted with ML29. Necropsies for 6 animals were performed on days 426, 452, 458, 491, 505, and 516 (after SIV). Five months after ML29 vaccination, 5 animals were still surviving. (X) indicates the relative position on the timeline of samplings and necropsies.

The experimental plan is diagrammed in Figure 1. Briefly, 393 days after infection with SIVmac251, eleven rhesus macaques were enrolled in this study. Eight macaques were inoculated with an attenuated LASV vaccine ML-29, in order to determine whether they could still elicit LASV-specific immune responses without developing signs of arenavirus disease. Five of the 8 were given ML29 subcutaneously (s.c.) and 3 were given ML29 intragastrically (i.g.). Both routes were successful, but the i.g. route required a higher dose [28]. Three additional SIV-infected macaques were inoculated with LCMV-Armstrong known from previous studies to cause a brief, uneventful infection in SIV-infected monkeys (MSS and JCZ unpublished). As controls, 5 healthy non-SIV-infected rhesus macaques were vaccinated s.c. with ML29.

**Figure 2:**
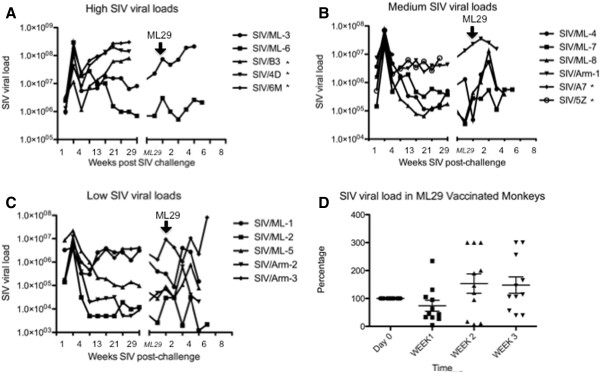
**Plasma SIVmac251 viral loads after infection.** Sixteen monkeys were classified based on the viral load peak after SIV infection as follow: (**A**) high, (**B**) medium, or (**C**) low. Viral loads were monitored up to 2 years after SIV infection. 7 months after (represented as a space in the X axis) eleven surviving monkeys were vaccinated with ML29 and SIV viral loads were reported up to 7 weeks. * denotes monkeys that died before ML29 vaccination. **D**) Percentage of variation in SIV viral loads is depicted over the first 3 weeks after ML29 vaccination. Seven of the 8 SIV/ML29 monkeys experienced a drop in SIV (median 20%) during the first week after vaccination, but those titers returned the following week.

SIV-positive animals were classified as slow, median or rapid progressors based on their physical signs and SIV viral loads at set point, i.e. 3 months after infection (Table 1, Figure 2A, B, and C). Seven of the 8 SIV/ML29 monkeys experienced a drop in SIV titers (median 20%) during the first week after ML29 inoculation, but those titers returned the following week (Figure 2D).

**Table 1:** Immune responses and viremia in SIV/ML29-infected monkeys and controls

**Animal ID**	**Cell-mediated immunity (IFNγ response) 1 week after vaccination**^**b**^	**Antibody response**^**c**^	**Plasma Viremia **^**d**^	**Time of euthanasia after ML29 **^**e**^	**SIV viral load at set point **^**f**^
SIV/ML-1 sc^a^	++++	++++	80 pfu/ml	Week 8	(M) 10^5^-10^6^
SIV/ML-2 sc	++	++++	----	>20 wks	(S) 10^3^-10^4^
SIV/ML-3 sc	++	+++	1000 pfu/ml	Week 4	(R) 10^7^-10^8^
SIV/ML-4 sc	++	++++	----	>20 wks	(M) 10^5^-10^6^
SIV/ML-5 sc	+++	+++	----	>20 wks	(S) 10^4^-10^5^
SIV/ML-6 ig	+++++	----	----	Week 14	(M) 10^5^-10^6^
SIV/ML-7 ig	----	----	----	Week 19	(M) 10^5^
SIV/ML-8 ig	+++	----	20 pfu/ml	>20 wks	(M) 10^5^-10^6^
SIV/ARM-1 sc	++	++	----	Week 16	(R) 10^7^
SIV/ARM-2 sc	++	++++	----	>20 wks	(S) 10^4^
SIV/ARM-3 sc	+++	+++	----	Week 9	(R) 10^6^-10^7^
ML-1 sc	+++	++++	----	>20 wks	----
ML-2 sc	+++	+++	----	>20 wks	----
ML-3 sc	++	+++	+	>20 wks	----
ML-4 sc	+++	+++	+	>20 wks	----
ML-5 sc	++	+++	----	>20 wks	----

^a^ SIV/ML-1 to SIV/ML-5 are macaques that were SIV-infected, then 393 days later, they were ML29-infected via a subcutaneous (sc) route. SIV/ML-6 to 8 were similarly SIV-infected, but then vaccinated with ML29 by an intragastric (ig) route. SIV/ARM-1 to 3 were SIV-infected and inoculated over a year later with LCMV-Armstrong by the subcutaneous route. Monkeys ML-1 through ML-5 were never SIV-infected and were all inoculated sc with ML29 only.

^**b**^ IFNγ responses for 8 SIV/ML animals, SIV/ARM animals and 5 ML29-vaccinated animals were followed up to 22 weeks. Primary arenavirus-specific responses were measured by IFNγ intracellular staining: maximum staining (+++++) corresponds to 0.4% of CD8+ cells, and minimum staining (+) corresponds to 0.012%. Since staining was negligible for CD4+ lymphocytes, measurements are only shown for CD8+ lymphocytes. (− − −) means non-detectable.

^c^ Arenavirus-specific antibody responses were measured by ELISA. The minimum and maximum optical density readings at 450 nm were 1.2 and 2.9 at 1:100 dilution, respectively. OD higher than 0.5 was considered positive.

^d^ Viremia in plasma of 3 ML29-vaccinated animals refers to the transient presence of infectious ML29 particles detected by conventional plaque assay. By sensitive 2-step co-cultivation assays, none of the other SIV-infected animals had detectable ML29 in plasma or urine. (+) in two non-SIV-infected monkeys means the plasma of these animals had co-cultivation-detectable ML29 in plasma (week 1 for ML-3 and week 2 for ML-4).

^**e**^ Time of euthanasia for those animals that lost more that 10% of body weight.

^f^ SIV viral loads. Animals were classified as Rapid AIDS progressors (R) with >10^6^ viral particles/ml; Medium, (M) between 10^5^-10^6^ viral particles/ml; and Slow (S) with <10^5^ viral particles/ml.

**Figure 3:**
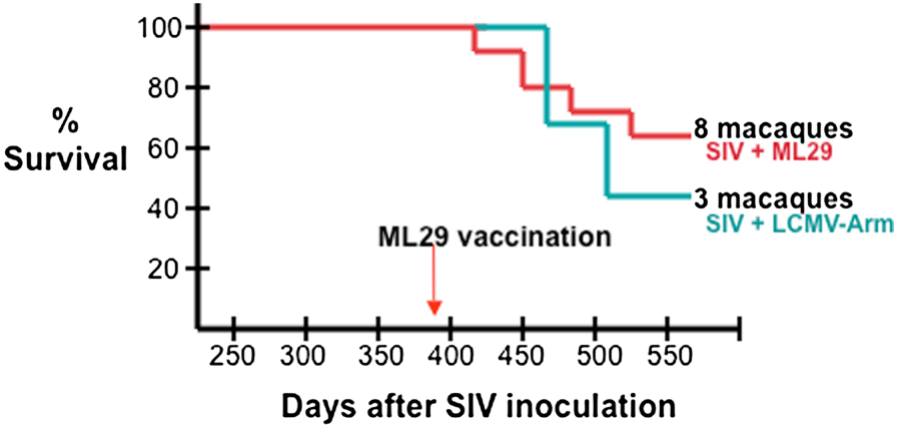
**Survival of macaques after SIV-infection and ML29 vaccination.** Eleven of 16 rhesus macaques that had been infected with SIVmac survived to be enrolled in a ML29 vaccination study (day 393 after SIV infection). Macaques were classified as rapid (>10^6^ SIV RNA mol/ml at setpoint), median (10^4^ – 10^6^ RNA mol/ml), and slow (<10^4^ RNA mol/ml) AIDS progressors. Eight were vaccinated with ML29 and three were given LCMV-Armstrong, and all were monitored for signs of arenavirus disease. Five months after vaccination, 5 healthy animals remained, having survived 530 days after SIV infection. The absence of gross signs of arenavirus disease and the absence of clinical signs in blood and tissues samples indicated that vaccinated animals were protected from arenavirus disease.

**Figure 4:**
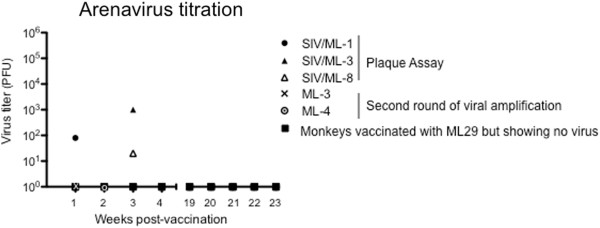
**Detection of arenavirus in ML29-inoculated macaques.** Virus titration by plaque assay revealed that only three animals (SIV/ML-1 •, SIV/ML-3▴, and SIV/ML-8 ▵) showed low viremia by week 3 after inoculation with ML29. Negative samples were tested by a second round of amplification (see Methods), showing traces of virus in two animals (ML-3 × and ML-4 ⊙), the remaining animals (▪) tested negative until the end of the study.

Two rapid-progressors were euthanized on days 34 and 63 and two median progressors were euthanized on days 57 and 105 after ML29 vaccination (Table 1, Figures 2 and 3). The first euthanized animal (SIV/ML-3) had high SIV loads and wasted appearance prior to ML29 vaccination. This animal developed a barely-detectable ML29 viremia (10^3^ pfu/ml of plasma) 3 weeks after vaccination. (*This titer of 10*^*3*^*pfu ML29/ml is still below the >10*^*4*^*pfu/ml considered to be a disease sign related to poor prognosis in LHF)*. Transient ML29 viremia (80 pfu/ml of plasma) was also detected one week after vaccination in the second euthanized monkey (SIV/ML-1), and in a long-term surviving monkey (SIV/ML-8 had 20 pfu/ml plasma) 3 weeks after vaccination (Figure 4).

ML29 plasma viremia was measurable by conventional plaque assay in 3 of 8 SIV-infected monkeys but not in non-SIV-infected monkeys (Table 1). When ML29 was not detectable by direct plaque assay or by RT-PCR, it could sometimes be detected by a 2-step amplification in which plasma or "infected" cells were co-cultured with Vero cells, then the media was harvested and subjected to conventional plaque assay. By this sensitive assay, ML29 was detected in plasma of 2 of 5 non-SIV-infected monkeys (ML-3 at week 1 and ML4-at week 2 after ML29 vaccination). Solid tissues of ML29-vaccinated monkeys (spleen, LN, brain, liver, kidney, heart, lung, adrenals) were all negative for ML29 by direct plaque assays.

Urine samples were collected from the monkeys weekly and assessed for virus shedding. Only two of the monkeys (SIV/ML-7 and SIV/ML-6) had detectable virus in urine during weeks two and three after ML29 inoculation respectively. The shed virus was not ML29 by plaque morphology or by detection arrays [29] which showed it to be Adenovirus 52, a virus that has been isolated previously from zookeepers [30,31] and can cause subclinical infection in non-human primates [32].

### Rationale for euthanizing animals in these studies

All four of the earliest-euthanized SIV-infected animals had wasting that began prior to ML29-inoculation. Upon necropsy it was evident they also had swollen ileo-caecal lymph nodes that are a hallmark of terminal AIDS [33]. Five of the original 16 SIV-infected monkeys died of AIDS prior to the onset of the ML29 vaccinations, and the time to death of the 11 remaining monkeys was not detectably influenced by the ML29 vaccination. Two major signs of virulent arenavirus disease and more specifically of lethal Lassa fever are high levels of liver enzymes and petechiae [6]. None of the ML29-vaccinated animals in this study showed high liver enzyme levels. All hematological and chemical parameters [urine analysis (BUN, creatinine), blood chemistry (glucose, cholesterol), blood cell counts, hematocrit, and erythrocyte sedimentation] were within normal ranges and no macaques developed clinical manifestations of arenavirus disease. This absence of arenavirus-specific pathology resembles previous ML29 trials in monkeys, marmosets and guinea pigs [10,12,13].

At the end of this study, approximately 5 months after the first ML29 vaccination, 5 animals (SIV/ML-2, SIV/ML-4, SIVML-5, SIV/ML-8, SIV/ARM-2) were still healthy, never having shown signs of arenavirus disease.

### ML29-specific humoral and cellular immune responses

**Figure 5:**
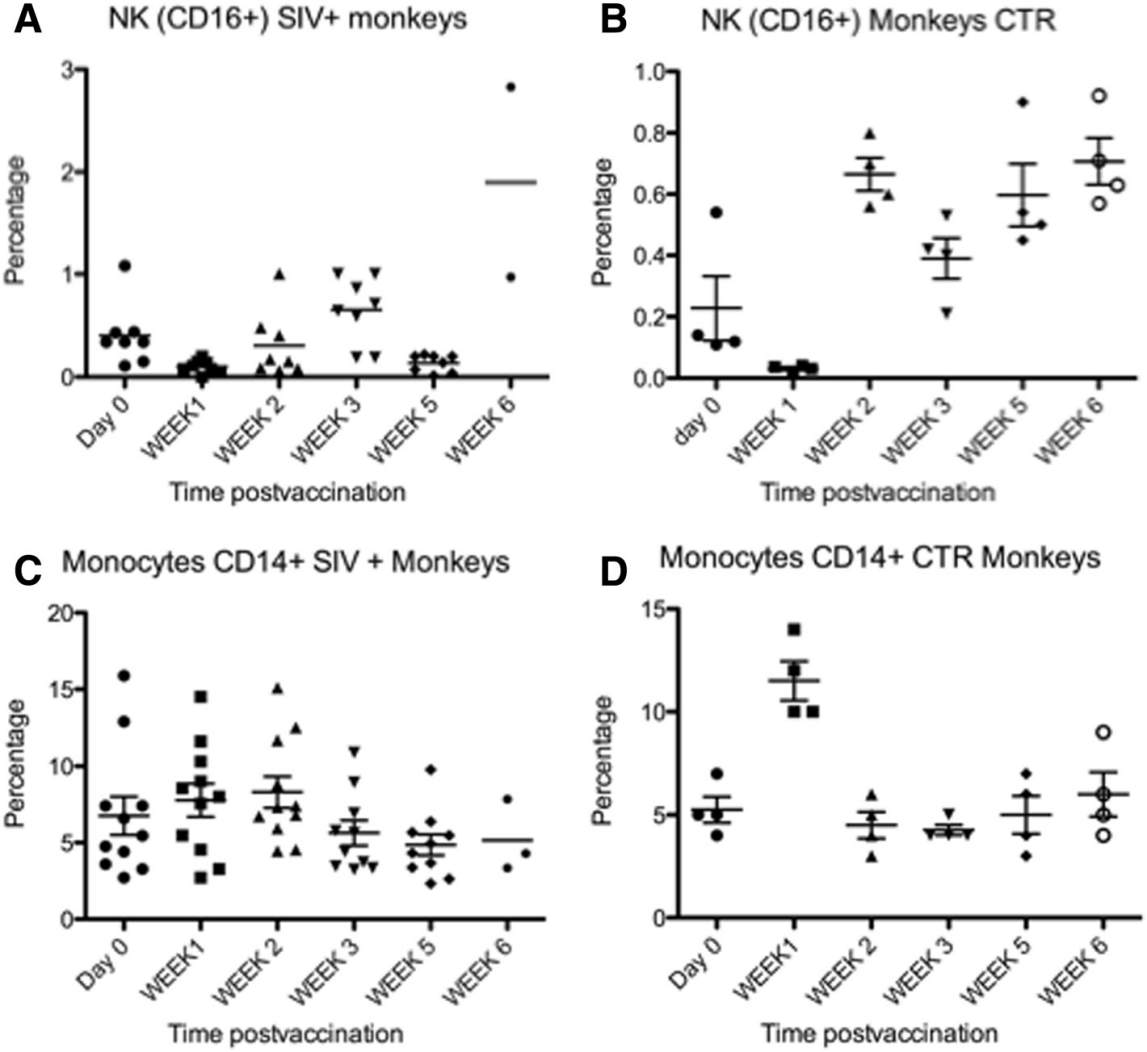
**Percentage of monocytes and NK cells in PBMC after ML29 vaccination. A**) In the 8 SIV-infected, ML29-vaccinated monkeys there is a significant reduction of the percentage of circulating NK (CD16+) cells one week after vaccination. **B**) Reduction of the NK (CD16+) population in four healthy (non-SIV-infected) animals one week after ML29 vaccination. **C**) In the 11 SIV-infected, arenavirus-vaccinated monkeys there is a slight increase in monocytes a week after ML29 vaccination. **D**) This increase is also observed in non-SIV-infected control monkeys.

In previous work we reported that rhesus macaques infected with the lethal LCMV-WE and non-pathogenic LCMV-Armstrong showed a drop in circulating NK and γδT cells, indicating that this is not a disease-specific event but instead infection-associated lymphopenia [34]. In agreement with this work, the analysis of blood cell subsets, in both SIV-infected and non-infected controls, showed a decrease in percentage of circulating NK (CD16+) cells a week after vaccination (Figure 5A and B). The CD14+ (monocyte) population showed a modest increase in the SIV-infected group and a marked increase in the control group one week after ML29 vaccination (Figure 5C and D).

Anti-LASV IgG antibodies were detected by ELISA from weeks 1 to week 5 and then monthly until the time of death. All animals showed good titers of anti-Lassa IgG except for those 3 given ML29 by the i.g. route. Optical densities (OD_450_) were observed to be higher than 1.25 for a 1:100 plasma dilution, when compared with negative controls at 0.30 OD_450_ (Table 1).

Cell-mediated immunity is believed to mediate LASV clearance from blood and tissues of immunized animals. Previously, we showed that a single s.c. ML29 immunization of rhesus macaques induced robust cell-mediated immune responses detectable in peripheral blood as early as 7 days after immunization [10]. Here the ML29-specific immune responses were measured by intracellular staining flow cytometry (ICS) for gamma-interferon (IFNγ) and confirmed by IFNγ ELISpot. High cell mediated immunity (++++) means that 0.4% of CD8+ T cells stained with intracellular IFNγ or that ELISpot levels exceded 200 positive cells per 10^6^. Low cell mediated immunity (+) means that 0.012% of CD8+ T cells stained with intracellular IFNγ or that ELISpot levels were less than 100 positive cells per 10^6^. All 5 control animals and seven of the 8 SIV-infected vaccinees had vigorous ML29-specific cell-mediated immunity by the first week after vaccination (Table 1). The animal with no ML29-specific cell-mediated response, (SIV/ML-7), also failed to produce anti-LASV IgG antibodies and survived 19 weeks after ML29 vaccination. When SIV/ML-7 finally succumbed, at a time consistent with median progression, it displayed the swollen ileo-caecal lymph nodes and increased SIV titer associated with AIDS [33]. Neither the ML-29 vaccine virus nor arenavirus disease symptoms were detected in SIV/ML-7. For those animals that survived longer than 16 weeks after ML29 vaccination, vaccine-specific cell-mediated immune responses lasted at least 18 weeks. The study was not designed to compare the longevity of immunity between SIV-infected and non-SIV-infected animals. Overall, cell-mediated immune responses were comparable in both groups although more variable in the SIV-infected monkeys.

### RNA profiling results

**Figure 6:**
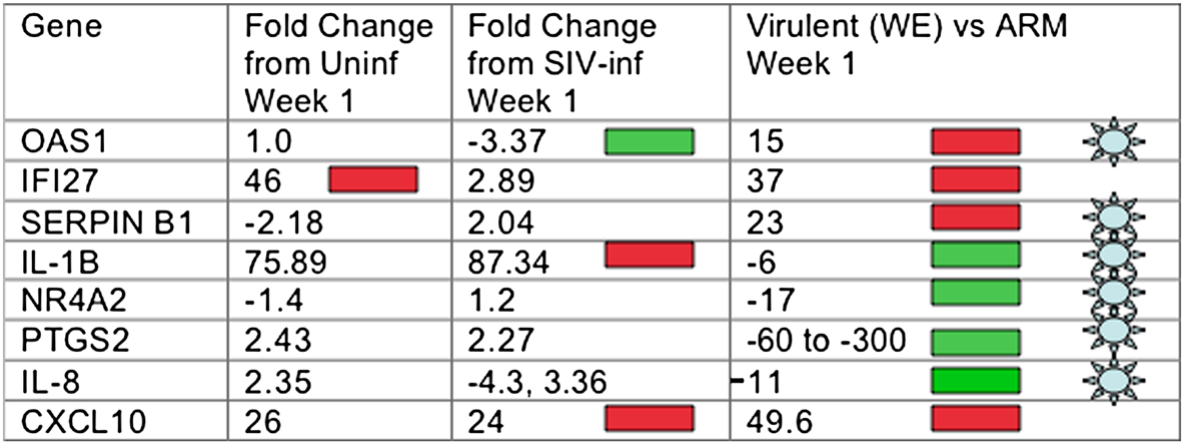
**Gene expression of SIV-infected monkeys given the ML29 Lassa vaccine.** Column one has the Genbank gene name. Column two has the fold difference in gene expression between ML29-vaccinated and uninfected (no SIV) monkeys, week 1. Column three has the fold difference in gene expression between ML29-vaccinated and SIV-infected, week 1. Column four has week one gene expression data from our previous profiling of monkeys inoculated with virulent and non-virulent strains of LCMV [35]. The blue star indicates indicates potential virulence markers in primate and guinea pig infections with virulent/mild virus pairs.

PBMC RNA was extracted 1 week after vaccination from SIV-infected and non-SIV-infected animals. Gene expression in macaque PBMC was determined by hybridization to Affymetrix microarrays as described in Methods. Approximately 100 genes were more than 2-fold differentially expressed in SIV/ML29-infected animals with respect to SIV-infection alone. The differentially-expressed genes were compared with our previously-published RNA profiles from LCMV-WE and LCMV-ARM infections in monkeys [35], that incidentally agree with RNA profiles of guinea pig infections with virulent and benign virus pairs [36-38]. We identified 8 genes: OAS1, IFI27, SERPIN B1, IL-1B, NR4A2, PTGS2 (the gene expressing cyclooxygenase 2), IL-8, and CXCL10 (the gene for cytokine IP-10) with potential to serve as virulence markers for Lassa-like arenavirus disease. The expression of these genes in the ML29-infected monkeys was more similar to their expression in non-pathogenic LCMV-Armstrong-infected monkeys than to their expression in LCMV-WE-infected monkeys that developed a Lassa-like disease (Figure 6).

## Discussion

The two goals of this study were first, to determine whether ML29 infection can be virulent or persistent during co-infection with SIV, and second, to determine whether ML29 is capable of eliciting robust Lassa-specific immunity in SIV-infected primates. In Lassa fever and in virulent LCMV-WE infection, viremia greater than 10^4^ pfu/ml plasma is correlated with bad prognosis [39,40]. Here, none of the ML29-vaccinated animals developed viremia greater than 10^3^ pfu/ml plasma. Nevertheless, SIV-infection resulted in more detectable ML29 virus than has been seen in uninfected macaques. In our previous studies with ML29-vaccinated macaques, no infectious ML29 virus could be detected in urine or plasma but traces of virus could be detected in lymphoid tissues only during the first two weeks after vaccination using sensitive two-step amplification techniques [10]. With ML29-vaccinated marmosets, only transient viremia was detected in 3 out of 16 animals by the two-step virus amplification technique, and viral nucleic acids could be detected in urine, saliva and vaginal swabs from only one animal [11]. With the SIV-infected monkeys in this study, the two-step amplification assay detected live ML29 virus in 3 of 8 ML29-vaccinated monkeys. Of the four SIV/ML29-monkeys still surviving after 20 weeks, two had experienced transient ML29 viremia and 2 had not; so the transient ML29 viremia did not have a measurable impact on health.

Next we determined whether the arenavirus ML29 established a chronic infection in the SIV-infected monkeys. In murine models of LCMV and LASV infection, immunological defects such as depleted CD4 or CD8+ T cells often resulted in viral persistence [41,42]. Arenavirus persistence is unusual in primates but long-term shedding of LASV has been observed [3], and high IgM titers against LASV antigens persist for months in Lassa patients [43]. In the case of SIV-infected monkeys, one might expect that an ordinarily transient ML29 infection could persist in the context of immunodeficiency. Monkey urine and plasma were monitored weekly, and ML29 virus was not detectable by five weeks after vaccination. Thus, the SIV-infection did not mediate any detectable virus persistence.

Classical signs of arenavirus disease include virus crossing the blood–brain-barrier, high liver enzyme levels in plasma, and petechiae. Virus was not detectable in brain tissue of any ML29 animals examined after necropsy. None of our ML29-vaccinated animals developed high levels of AST, ALT, or bilirubin, whereas in Lassa fever and in lethal LCMV-WE infection, AST greater than 150 IU/ml plasma indicates poor prognosis [39,40,44]. Petechiae typical of virulent arenavirus disease were not observed during daily monitoring of the ML29-vaccinated animals. Thus, ML29 did not become virulent in the context of SIV infection.

Non-classical signs of virulence were sought by transcriptome profiling of monkey PBMC-derived RNA. In our previous studies, extensive transcriptome profiles of rhesus macaques with virulent and mild arenavirus infections revealed gene expression that distinguished arenavirus infection from no infection, and gene expression that distinguished virulent infection from mild infection [35,45]. Transcriptome profiling of the SIV-infected rhesus macaques in this study indicated that all the animals were infected with an arenavirus, and none of them had gene-expression patterns consistent with virulent arenavirus infection. Comparison of data from SIV-infected cohorts with data from SIV/ML29-infected cohorts revealed differential expression of specific pathways, including the cyclo-oxygenase/prostaglandin (PTGS2) pathway, the Serpin B-related coagulation pathway, the nuclear-receptor 4 signaling pathway, interferon-stimulated genes (ISG) like OAS and IFI27, and the IL-8/IP-10 chemokines. Studies described here were consistent with the observation that SIV/ML29-infected animals did not display signs of gene expression previously associated with virulent arenavirus infections.

A second goal of our studies was to determine whether SIV/ML29-infected animals could elicit immune responses comparable to those seen in animals given ML29 alone. All but one of the 11 SIV-infected, arenavirus-infected animals elicited measurable cell-mediated and humoral immune responses and the levels were similar to those in the non-SIV-infected cohort. The one animal with no measurable immunity was a median AIDS progressor that lived until 19 weeks after ML29 vaccination when he succumbed to AIDS. Cell-mediated responses were variable in the SIV-infected cohorts but comparable to the ML29-alone cohorts, and ML29-specific antibody responses were frequently better in SIV-infected monkeys than in the non-SIV-infected monkeys. In a study conducted in LASV-infected cynomolgus monkeys [5] LASV-specific IgG responses were detected 12 days after challenge, with higher titers in survivors than in fatal cases. This suggests that the humoral response is induced earlier and more strongly in nonfatal infections [5]. We failed to observe a correlation between ML29 titers and survival, probably because survival in our study was due to AIDS progression and not related to the arenavirus infection.

Several other studies have used non-human primate AIDS models to explore the safety and efficacy of vaccines. In one study, SHIV-infected rhesus macaques were given a vesicular stomatitis virus-based ebola vaccine and all six vaccinees survived a lethal ebola challenge [17]. In another study SIVmac251-infected rhesus macaques were vaccinated against smallpox: those with low CD4 counts failed to elicit neutralizing antibodies and succumbed to lethal monkeypox challenge, whereas those with normal CD4 counts elicited neutralizing antibody and survived a lethal challenge [18].

In our study all the monkeys maintained normal CD4 counts but exhibited other signs of AIDS including SIV-viremia, wasting and lymphadenopathy. In fact 5 of the 16 SIV-infected monkeys scheduled for ML29 vaccination died of AIDS before they could be vaccinated. Attrition of the remaining 11 animals continued at a rate expected for death from AIDS alone; all dying macaques showed signs of AIDS rather than signs of arenavirus disease.

By the end of our study, 5 monkeys remained healthy without developing classical or non-classical signs of virulent arenavirus disease.

## Conclusions

SIV-infected macaques given the attenuated Lassa vaccine ML29 did not show increased signs of arenavirus disease or shortened lifespan. A slight increase in ML29 viremia in SIV-infected as compared to non-SIV-infected macaques was noted. No increased shedding or persistence of ML29 was noted in the SIV-infected hosts. ML29 elicits cell-mediated and humoral immune responses to LASV similarly in SIV-infected and non-SIV-infected animals.

## Materials and methods

### Viruses and cells

Mopeia/Lassa reassortant virus (clone ML29) was previously tested as a vaccine against Lassa fever [10,26,27]. Vero E6 cells (#CRL-1586, ATCC) were cultured in Dulbeccos’s modified Eagle’s medium (DMEM, GIBCO-BRL) supplemented with 10% fetal bovine serum (FBS, GIBCO-BLR), 1% penicillin-streptomycin, and 2 mM L-glutamine. To produce the ML29 inoculum, Vero cell monolayers were infected with ML29 master stock at a multiplicity of infection (MOI) of 0.01 and incubated 1 hour at 37°C in 5% CO_2_, then washed with PBS and covered in DMEM 2% FBS. Supernatants were collected at 48 and 72 hours after infection, stored at -70°C, and titrated on Vero cells. Supernatants usually had about 10^7^ plaque forming units (pfu)/ml. Stocks of lymphocytic choriomeningitis virus-Armstrong (LCMV-ARM) and LCMV-WE (described extensively in [44]) were similarly produced and titrated on Vero E6 cells and stored at -70°C.

SIVmac251 (originally supplied by Ron Desrosiers) was amplified on rhesus macaque PBMC and given a final passage on CEMX174 cells as described [46]. Monkeys were infected by inoculating 10 TCID50 per animal by the saphenous vein.

Whole blood collected in heparin was used to stain for cell surface and intracellular markers (especially for cell-mediated immunity assays). Fresh monkey PBMC for transcriptome analyses were obtained from 10 ml of heparin blood by Ficoll-hypaque isolation [47] and resuspended in RPMI medium 1640 with 10% FBS (Sigma) for RNA extraction as described below [35]. Residual PBMC were frozen at -140°C at a concentration of 10^7^ cells/ml. Upon necropsy, splenocytes were obtained for cell-mediated immunity assays: fresh tissue pieces were pressed through mesh, exposed to red cell lysis buffer 5 minutes, then suspended in freezing media and stored at −140°C at a concentration of 10^7^ cells/ml. Serum samples and plasma samples were stored at −140°C before being analyzed for virus or antibody content.

### Animals and immunization

Rhesus macaques (Rh) were purchased from the Caribbean Primate Colony. Experimental protocols were approved by the Institutional Animal Care and Use Committee at the University of Maryland School of Medicine. Of 16 monkeys inoculated with SIVmac251 as previously described [19], 5 were dying with symptoms of SIV disease and had to be euthanized almost a year after SIV infection and before the onset of the ML29 vaccinations. Of the remaining 11 animals, 8 were inoculated with the ML29 vaccine candidate and 3 were inoculated with LCMV-Armstrong, known to be benign in AIDS monkeys (Salvato MS and Zapata JC, not shown). Of the 8 ML29-vaccinees, 5 were inoculated s.c. with 0.5 ml 10^3^ pfu ML29 and 3 were inoculated i..g. with 2 ml 10^6^ pfu ML29. In addition, 5 healthy control animals were vaccinated s.c. with 0.5 ml of ML29 containing 10^3^ pfu. Blood was collected every week for flow cytometry, viral loads, antibody detection, clinical chemistry, hematology, and transcriptome profiles as described [35]. Six weeks after their first vaccination, 6 macaques (ML-1, SIV/ML-1, SIV/ML-2, SIV/ML-6, SIV/ML-7, SIV/ARM-1) were boosted s.c. with 10^3^ pfu ML29. Animals were monitored daily for weight loss, rashes and clinical signs of SIV or arenavirus disease.

For weight loss over 10% of body weight and in consultation with the veterinary staff, monkeys were euthanized and total blood and tissues [lung, spleen, mesenteric lymph nodes (LN), liver, stomach, ileum, kidney, heart, cerebrum, and cerebellum] were collected. A portion of each tissue was submerged in MEM with 10% FCS for virus and RNA isolation. The remaining tissue portions were flash frozen in liquid nitrogen to be used in virus isolation or fixed in 10% neutral formalin for the preparation of histological sections. Also, at the end of the study surviving healthy animals were euthanized and blood and tissues collected.

### Detection of SIV and arenavirus in monkey tissues

SIV viral loads were evaluated from plasma samples using real-time NASBA to determine the number of RNA copies per milliliter (Advanced Biosciences Laboratory, Kensington, MD). This technique can detect 100 SIV RNA copies per ml plasma. Based on SIV-infected animal set points [48,49], monkeys were considered slow AIDS progressors (<10^4^ RNA molecules/ml), median progressors (10^4^ to 10^6^ RNA molecules/ml), and rapid progressors (>10^6^ RNA molecules/ml). Of the eight SIV-infected animals inoculated with ML29, two were slow progressors (SIV/ML-2, SIV/ML-5), 5 were median progressors (SIV/ML-1, SIV/ML-4, SIV/ML-6, SIV/ML7, and SIV/ML-8) and one was a rapid progressor (SIV/ML-3). Of the three monkeys inoculated with the mild LCMV-ARM, one was a slow progressor (SIV/ARM −2) and two were rapid progressors (SIV/ARM −1 and SIV/ARM −3).

Arenaviruses were detected by three different methods: 1) conventional plaque assays that are sensitive to 20 infectious particles in 1 ml solution and were used for assessments of plasma or tissue viremia for arenaviruses ML29, LCMV-Armstrong, and LCMV-WE [11]; 2) RT-PCR that detects 10 viral genomes in 200 μl serum and was used in all tissues samples (as described in [11]; and 3) a sensitive 2-step amplification assay in which infected plasma or cells are co-cultivated with Vero cells one week, then the media is subjected to plaque assay. The 2-step assay increases the plaque detection up to 1 infectious particle per ml [10,11].

### Flow cytometry to characterize PBMC

Ten ml of whole blood was collected in heparin tubes, and 100 μl was mixed with each antibody panel (mixtures of fluorochrome-conjugated antibodies supplied by BD Bioscience San Jose, CA). Panel 1 included mouse anti-human CD20-FITC (clone 2H7), CD3-PE (clone SP34-2), CD4-APC (clone L200), and CD8-PerCP (clone SK1) to detect B and T lymphocyte populations. Panel 2 included mouse anti-human CD16-FITC (clone 3 G8), CD56-PE (clone NCAM16.2), CD4-APC (clone L200), and CD8-PerCP (clone SK1) to detect NK, gamma-delta T cell (γδT cell), and NKT cell populations. Panel 3 included CD14-FITC (clone M5E2) and CD4-APC (clone L200), to detect monocyte and T cell populations. Panel 4 included CD3-PE, and TCR Vgamma9-FITC (clone IMMU 360, Beckman Coulter). After 30 min, FACS Lysing Solution (BD) was added for red cell lysis and incubated 20 min at room temperature. Cells were washed twice with PBS and fixed in 2% formaldehyde, then collected in a FACScalibur (BD) and data were analyzed with FlowJo Software (Tree Star, San Carlos, CA).

### **Intracellular staining for virus-specific IFN**γ **responses**

Whole heparinized blood (0.5 ml) was stimulated with <50 μl of ML29 (5 × 10^5^ pfu) or MEM (control) at 37°C, 5% CO_2_ overnight. 25 μl of 1:10 dilution of monensin (Golgiplug, BD) was added to each sample 4 hours prior to staining. Then cells were stained for surface antigens with anti-CD4-APC (clone L200) and CD8 (clone SK1) and stained for intracellular IFNγ using monoclonal antibody 4S.B3 and the Cytofix Cytoperm kit (BD). Samples were analyzed on a FACSCalibur instrument with 50,000 events in the lymphocyte gate and results were analyzed using FlowJo software (TreeStar).

### IFNγ ELISPOT

PBMC from immunized animals were used in IFNγ ELISPOT (U-CyTech B.V., Ultrecht, The Netherlands) according to the manufacturer's recommendations with slight modifications [10]. Briefly, 2 × 10^6^ cells in 0.5 ml of RPMI-1640 (Invitrogen) with 5% FBS*,* 2 mM glutamine, 100 units/ml penicillin, 100 μg/ml streptomycin, and 25 mM Hepes buffer were stimulated by co-incubation overnight at 37°C with 2 × 10^6^ pfu of ML29. After stimulation, the cells were washed, resuspended in the same medium, and 0.3–0.4 × 10^6^ cells/well were added to ELISPOT 96-well plates pre-coated with mouse anti-monkey IFNγ. The plates were incubated at 37°C for 5 h, washed, and incubated with gold-conjugated anti-biotin. The spot-forming cells (SFC) secreting IFNγ were developed with activator solution and counted (Immunospot 3.2 Analyzer, C.T.L. Cellular Tech., Ltd.)

### ELISA

Anti-LAS-GPC antibodies in serum samples were measured by IgG ELISA as previously described [10]. Supernatants of ML29-infected Vero E6 cells were concentrated in 15 ml Amicon tubes then sonicated using a Misonix-S4000 sonicator (MISONIX, Newtown, CT). This concentrated-antigen was suspended in carbonate-bicarbonate buffer (pH 9.6), and 100 μl of antigen was adsorbed to the wells of microtitration plates overnight at 4°C. After washing the 96-well plates 6 times with PBS-Tween (0.05%), two-fold dilutions (1/50 to 1/400) of plasma were added and incubated for an hour at 37°C. Wells were washed 5 times, and 100 μl of 5,000-times diluted peroxidase-conjugated goat anti-monkey IgG (A-2054, Sigma) was added and incubated one hr, then washed 6 times, then substrates were added, incubated for 30 min in the dark, then stopped with 100 μl/well of 1 M phosphoric acid and read at OD_450_ on a Wallac 1420 plate-reading spectrophotometer.

### Gene expression from monkey PBMC cDNA

Total RNA was isolated from fresh PMBC samples, using the Trizol method (Invitrogen, Carlsbad, CA) followed by a cleaning step with RNeasy mini kit (Qiagen, Valencia, CA). Quality and quantity of all RNA samples were evaluated on an Agilent 2100 BioAnalyzer 116 (Agilent Technologies, Palo Alto, CA) by looking at 18 and 28 s rRNA peaks and by the RIN (RNA integrity number). High quality RNA was labeled and hybridized according to Affymetrix protocols using the human GeneChip U133 Plus 2.0 array (Affymetrix, Santa Clara, CA) as described previously [45]. This chip covers the whole human genome using 54,000 probesets representing approximately 22,000 genes and has been validated for use with non-human primates [50-52]. Although many PBMC-RNA samples were analyzed, the only ones from sufficiently large groups to yield statistically significant data were the SIV-infected samples (n = 8), the SIV/ML29 s.c. week 1 samples (n = 5) and the SIV/ML29 s.c. week 2 samples (n = 5). Smaller groups included SIV/ML29 i.g. weeks 1 and 2, SIV/LCM s.c. weeks 1 and 2, ML29 i.v. weeks 1 and 2 and the uninfected samples.

## Abbreviations

SIV: Simian immunodeficiency virus; ML29: Mopeia Lassa reassortant isolate 29; LASV: Lassa virus; LCMV: Lymphocytic choriomeningitis virus (including strains LCMV-Armstrong and LCMV-WE); Pfu: Plaque forming units for arenavirus titers; IFNγ: Interferon-gamma; s.c: Subcutaneous; i.g: Intragastric; i.v: Intravenous; LN: Lymph nodes; NASBA: Nucleic Acid Sequence Based Assays; RT-PCR: Reverse transcription followed by polymerase chain reaction.

## Competing interests

The authors declare that they have no competing interest.

## Authors’ contributions

JCZ coordinated this work, carried out laboratory experiments, data analysis and drafted the manuscript; BP, JB, HD, EA, and LG did all animal vaccinations, sample collections and reviewed the manuscript; JCZ, TS, and CJ, participated in virus identification from urine samples; OC and YZ performed the microarray analyses of monkey PBMC RNA; MG and JCZ did immune assays; DM and JCZ detected gene expression and genetic integration of ML29; CDP provided animals and helped to draft the manuscript; ISL, helped to interpret results; MSS conceived, designed and helped to draft the manuscript. All authors read and approved the final manuscript.
